# Study of the Influence of Melamine and Expanded Graphite on Selected Properties of Polyurethane Foams Based on Uracil Derivatives

**DOI:** 10.3390/polym17192610

**Published:** 2025-09-26

**Authors:** Elżbieta Chmiel-Szukiewicz, Joanna Paciorek-Sadowska

**Affiliations:** 1 Department of Organic Chemistry, Faculty of Chemistry, Rzeszow University of Technology, al. Powstańców Warszawy 6, 35-959 Rzeszów, Poland; 2 Department of Chemistry and Technology of Polyurethanes, Institute of Materials Engineering, Kazimierz Wielki University, J. K. Chodkiewicza Street 30, 85-064 Bydgoszcz, Poland

**Keywords:** polyurethane foams, melamine, expanded graphite, flame retardant

## Abstract

Polyurethane foams containing heterocyclic rings are characterized by high thermal resistance, but unfortunately, they are flammable. This work examined the effect of halogen-free flame retardants such as melamine and expanded graphite: EG 096 and EG 290 on the properties of foams with a 1,3-pyrimidine ring. Oligoetherol obtained from 6-aminouracil, ethylene carbonate, and propylene oxide was foamed with polymeric diphenylmethane 4,4′-diisocyanate with the addition of flame retardants. The oxygen index was determined, and flammability tests were conducted on the resulting foams. Their apparent density, water absorption, thermal resistance, thermal conductivity coefficient, and compressive strength were also examined. Both melamine and expanded graphite significantly reduce the flammability of foams. The resulting foams are classified as V-0 flammability class, and their oxygen index is in the range of 24.9–29.5 vol.%. Expanded graphite is a better flame retardant and does not cause deterioration of other foam properties.

## 1. Introduction

Polyurethane foams are formed by the polyaddition reaction of multifunctional isocyanates with polyols in the presence of foaming agents, catalysts, and surfactants [1]. Depending on their apparent density and strength, such materials are classified as rigid, semi-rigid, and flexible. These properties are mainly dependent on the structure of the polyol component [1]. Polyurethane foams are currently widely used in many industries, primarily construction, furniture, and automotive. Rigid polyurethane foams are primarily used as insulating materials (for thermal and acoustic insulation and foundations), so it is important that they have good thermal resistance, low thermal conductivity, low water absorption, and are non-flammable. The flammability of these materials is reduced by introducing flame retardants, such as organic compounds of boron [2,3,4,5,6,7] or phosphorus [8,9,10,11,12]. The flame retardants commonly used in polyurethane foams are also melamine and its derivatives, such as phosphate and isocyanurate [12,13,14,15,16]. Melamine’s flame-retardant effect is that at high temperatures it condenses to melam (∼350 °C), melem (∼450 °C), and melon (∼600 °C). The resulting condensates form a charred layer that isolates the foam surface from flames. Furthermore, ammonia released during condensation is a non-flammable gas, which prevents the flame from being sustained, especially in the vicinity of the developing charred layer [17]. Another relatively inexpensive, effective, and environmentally friendly flame retardant is expanded graphite [17,18,19,20,21,22,23,24,25]. Graphite expands under the influence of heat, forming a swelling layer on the foam’s surface. This slows the spread of fire and prevents the formation of toxic gases and smoke. Furthermore, the charred layer that forms on the surface of the burning foam protects the unburned part from heat and oxygen [17,20,25]. This prevents the spread of fire across the foam surface in the part not yet affected by the flame and consequently gives the foam self-extinguishing properties. Expanded graphite with larger particle sizes undergoes greater expansion after heat treatment, resulting in the formation of a sufficiently thick char layer on the surface of the composite to ensure its non-flammability [21,25]. However, the use too much of expanded graphite as a flame retardant causes deterioration in the mechanical properties and thermal conductivity of polyurethane foams. Therefore, it is important to select the optimal amount of this flame retardant [17,20]. Halogen-free flame retardants are currently being used in various industries, replacing halogen-based additives. In addition to the aforementioned polyurethane foams, halogen-free flame retardants are also used in wire and cable construction [26], for the production of flame-retardant textiles [27] or fire-resistant coatings [28].

The use of hydroxyalkyl derivatives of 6-AU (I) as a polyol component in the synthesis of polyurethane foams allows for polyurethane foams with high thermal resistances (up to 200 °C) to be obtained [29]. The disadvantage of these materials is their flammability, which significantly limits their use, for example, as insulating materials.


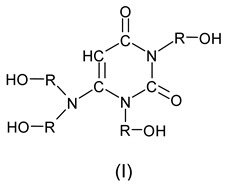


Reducing the flammability of foams by introducing boron atoms into the structure of oligoetherols with a 1,3-pyrimidine ring resulted in deterioration of thermal resistance and increased water absorption of foams [5,6]. Therefore, the introduction of a flame retardant by the additive method during the foaming process seems to be a better solution. In this study, it was decided to use melamine and expanded graphite to reduce the flammability of foams with a 1,3-pyrimidine ring.

## 2. Materials and Methods

### 2.1. Materials

The following materials were used in this work: 6-aminouracil (6-AU, pure 98%; AmBeed Inc., Arlington Heights, IL, USA), ethylene carbonate (EC, pure ≥ 99%, Merck, Darmstadt, Germany), potassium carbonate (anal. grade 100%, POCH, Gliwice, Poland), propylene oxide (PO, technical ≥ 99%, VWR International S.A.S., Rosny-sous-Bois, France), triethylamine (TEA, anal. grade ≥ 99%, Fluka, Buchs, Switzerland), polymeric diphenylmethane 4,4′-diisocyanate (p-MDI, Merck, Darmstadt, Germany; %NCO = 32.8), surfactant Silicon L-6900 (pure, Momentive, Wilton, CT, USA), melamine (pure 99%, Sigma-Aldrich, Steinheim, Germany), expanded graphite 096, (EG 096, average particle size: 100 μm; expansion rate: 40–80 mL/g, Sinograf S.A., Toruń, Poland), and expanded graphite 290 (EG 290, average particle size: 400 μm; expansion rate: 200–300 mL/g, Sinograf S.A., Toruń, Poland).

### 2.2. Oligoetherol Preparation

Oligoetherol was obtained from 6-AU, EC, and PO in the molar ratio 1:5:3, according to the procedure described in work [29].

To a 250 cm^3^ round-bottomed flask equipped with a stirrer, reflux condenser, and thermometer, 12.7 g (0.1 mol) of 6-AU, 44.0 g (0.5 mol) of EC, and 0.4 g of potassium carbonate as a catalyst were introduced. The mixture was heated to 140 °C with continuous stirring. The end of the reaction was established by determining the amount of unreacted EC. A resin-like product was obtained in this reaction. Next, 34.7 g (0.1 mol) of the reaction product 6-AU with 5 moles EC, 17.4 g (0.3 mol) of PO, and 1 cm^3^ of TEA as a catalyst were introduced into a 250 cm^3^ autoclave equipped with a stirrer. The mixture was heated to 50 °C, vigorously stirred until a homogeneous system was obtained, and then maintained at a temperature of 60–70 °C under pressure of up to 2 atmospheres (2026.5 hPa). The end of the reaction was established by determining the epoxy number of the reaction mixture.

### 2.3. Foams Preparation

In a 500 cm^3^ cup, 10 g of oligoetherol obtained from 6-AU, EC, and PO were weighed, then 0.3 g of water (foaming agent), an appropriate amount of silicone (surfactant), TEA (catalyst), and melamine or expanded graphite EG 096 or EG 290 (flame retardant) were added (Table 1). The mixture was thoroughly stirred. After homogenization, 14.4 g of p-MDI was added and stirred until creaming began. Foaming was performed at room temperature. The obtained foams were seasoned for at least 48 h at room temperature and then samples were cut out for further tests.

### 2.4. Analytical Methods

The amount of unreacted EC was determined by the barium hydroxide method described in work [30]. Epoxy number was determined by titration of a sample with hydrochloric acid in a dioxane solution [31]. The pictures of the cross-sectional areas of the foams were taken using a Pantera microscope (prod. Motic, Wetzlar, Germany) with 4 × 10 lenses and worked up with Motic Multi-Focus Professional 1.0 software, enabling the merging and manipulation of images with an adjustable lensing plane. The apparent densities of the foams were calculated for cube-shaped samples according to the norm [32], as the ratio of the foam mass to its geometrical volume. Water absorption in vol.% of the foams was determined according to the norm [33], by immersing the samples in distilled water and measuring the mass after 5 min, 3 h, and 24 h. Linear dimension stability of foams was tested according to the norm [34], by thermostating the samples at a temperature 150 °C for 20 and 40 h and measuring changes in the length, width, and thickness of the samples. Thermal resistances of the foams were determined by the static method: the foams were heated for a month at 150, 175, and 200 °C with continuous measurement of mass loss. The thermal analysis of foams (dynamic method) was conducted with a Thermobalance TGA/DSC 1 derivatograph (Mettler Toledo, Greifensee, Switzerland); the recording conditions were as follows: sample weight of 2–4 mg, temperature range of 25–800 °C, heating rate of 10 °C min^−1^, recording time of 60 min, nitrogen atmosphere. The thermal conductivity coefficient was measured with IZOMET 2104 (Bratislava, Slovakia) according to the norm [35]. The compressive strengths at 10% strain (σ_10_) of the foams were tested according to the norm [36] using the Instron 5967 strength testing machine (INSTRON 5967, Grove City, PA, USA). The maximum force inducing 10% relative strain of the foams (decreasing height of the foams in relation to the initial height, in accordance with the direction of foam rise) before and after exposition at 150, 175 and 200 °C was determined. Oxygen index was measured with Concept Equipment apparatus (Concept Equipment, Rustington, UK) according to the norm [37], for 150 × 10 × 10 mm samples. The horizontal burning test was performed according to the norm [38], for 150 × 50 × 13 mm samples. The sample was placed on a stainless-steel net and set on fire from the bottom. Then the burner was removed and the free-burning time of the foam was measured from when the flame or glowing combustion front passed the 25 mm gauge mark from the edge sample. Then, the following were also determined: the distance burnt (between the 25 mm gauge mark and the point where the flame or the glowing combustion front stopped) [mm], the flame rate ν [mm/s], and mass loss. Vertical flammability tests of the foams were performed according to the norm [39], for 127 × 13 × 13 mm samples. The vertically positioned sample was set on fire from the bottom twice for 10 s. Each time, the burning time of the foam was measured after the burner was removed. That was the way to classify the foam flammability class.

## 3. Results and Discussion

### 3.1. Oligoetherol Synthesis

**Scheme 1:**
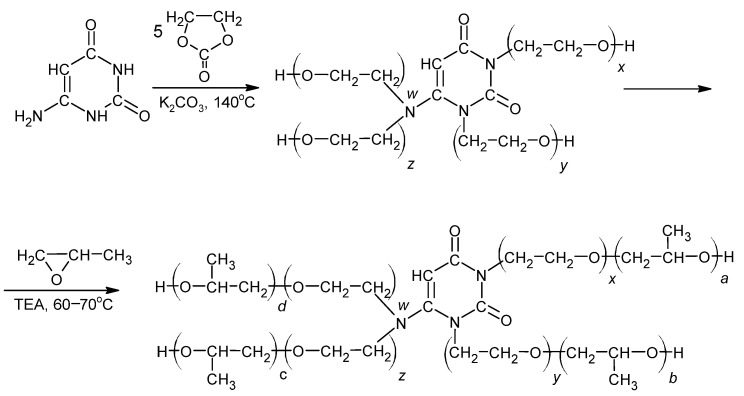
Reaction of 1 mol 6-AU with 5 moles EC and 3 moles PO; where x + y + z + w = 5 and a + b + c + d = 3.

The work was started by obtaining the polyol agent by reaction of 1 mole of 6-AU with 5 moles of EC and 3 moles of PO (Scheme 1), according to the procedure described in [29]. The synthesis takes place in two stages. First, 6-AU is reacted with EC in the presence of potassium carbonate as a catalyst at a temperature of 140 °C, and then the obtained intermediate is transferred to a pressure reactor and reacted with PO in the presence of TEA at a temperature of 60–70 °C [26]. The structure and properties of oligoetherol are described in [29].

### 3.2. Polyurethane Foams Synthesis

Polyurethane foams were synthesized using the obtained oligoetherol, polymeric diphenylmethane 4,4′-diisocyanate, TEA as a catalyst, water as a blowing agent, silicone as a surfactant, and melamine or expanded graphite as a flame retardant. Foaming was carried out on a small laboratory scale, and the composition of foaming samples was selected experimentally, based on previous studies [5,6,29]. The best foams were produced when 144 g of p-MDI, 3.5 or 3.84 g of silicone, from 0.27 to 0.94 g of TEA, and 3 g of water were used per 100 g of oligoetherol (Table 1). In the compositions, the mass addition of flame retardant was changed, 100 g of melamine, 60–80 g of EG 096, and 40–60 g of EG 290 were added per 100 g of oligoetherol (Table 1). The amount of flame retardant used was limited by the difficulty of good component homogenization and the possibility of non-uniform distribution within the foam structure. Introducing a smaller amount of a given flame retardant did not give a satisfactory oxygen index result. All the obtained foams were rigid.

EG 096, characterized by a powdery structure and a grain size of 0 to 0.15 mm, homogenized well with the foaming ingredients, and foams with this addition had a uniform gray-beige color, without visible individual graphite particles. As the percentage of this graphite increased, the foam developed became an increasingly darker graphite color. EG 290 has a flake structure, with a grain size of 0.2–0.6 mm; therefore, the maximum amount that could be introduced into the composition was 60 wt.% in relation to the weight of the oligoetherol. Dispersed flame-retardant particles were visible in the foam structure with the EG 290 addition.

**Figure 1:**
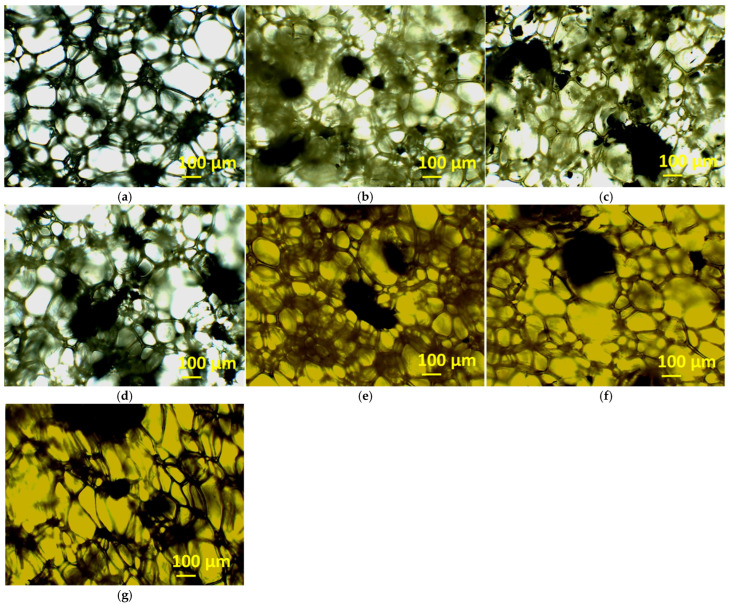
Microscope image of the foams: composition no. 1 (**a**), composition no. 2 (**b**), composition no. 3 (**c**), composition no. 4 (**d**), composition no. 5 (**e**), composition no. 6 (**f**), and composition no. 7 (**g**).

The cross-sectional photos of the synthesized foams show that they are characterized by oval pores (Figure 1a–g). The foam with the melamine addition had pore dimensions (larger diameter x smaller diameter) from 55 × 43 µm to 270 × 182 µm (Figure 1a), that with 60% EG 096 from 62 × 61 µm to 128 × 81 µm (Figure 1b), with 70% EG 096 from 47 × 35 µm to 157 × 107 µm (Figure 1c), with 80% EG 096 from 59 × 38 µm to 166 × 133 µm (Figure 1d), with 40% EG 290 from 45 × 42 µm to 180 × 131 µm (Figure 1e), with 50% EG 290 47 × 39 µm do 181 × 145 µm (Figure 1f), and with 60% EG 290 from 62 × 32 µm to 278 × 82 µm (Figure 1g). The largest pore size distribution and the largest pores are found in the foam with melamine added as a flame retardant (Figure 1a). Foams with expanded graphite added are characterized by smaller pores, and as the amount of this flame retardant increases, the pore size increases (Figure 1b–g). Classic rigid polyurethane foams usually have pore diameters of 200–600 μm [40], so it can be concluded that the compositions obtained are characterized by relatively small pores.

### 3.3. Properties of Foams

The apparent density of the synthesized foams ranged from 51 to 76 kg/m^3^, higher than that of the composition without flame retardant (Table 2). The foam with melamine added had the highest density, while the foam with EG 290 added had the lowest. In the case of using EG 096, the apparent density decreased with increasing flame-retardant addition; the amount of EG 290 did not have an effect on the apparent density of the composition (Table 2). Foams with boron atoms as flame retardant had a higher apparent density, ranging from 66 to 91 kg/m^3^ [6,7].

The effect of the addition of flame retardant on water absorption was then tested. The obtained compositions were characterized by very low water absorption, reaching a maximum of 4.5 vol.% after 24 h of exposure to water (Table 2), suggesting the presence of closed pores in the materials. It can be seen that with increasing expanded graphite content in the foam structure, the amount of water absorbed decreased, and the composition containing 80% of this flame retardant demonstrated the lowest water absorption (Table 2). The obtained compositions were characterized by significantly lower water absorption than the foam without flame retardant (Table 2). Foams with boron atoms as a flame retardant had significantly higher water absorption, reaching up to 30 vol.% after 24 h of exposure to water [6,7].

The thermal conductivity coefficient of the obtained materials is in the range of 0.028–0.037 W/m·K and is at the upper limit of the range for rigid polyurethane foams. The lowest thermal conductivity coefficient (0.028 W/m·K) was characterized by composition 5, containing the smallest amount of graphite (40% EG 290, Table 2). With increasing content of this flame retardant in the composition, the conductivity coefficient increases (Table 2), but no significant increase in thermal conductivity is observed due to the addition of graphite. The tested foams have lower values of thermal conductivity coefficients than foams containing boron atoms as a flame retardant (0.035–0.041 W/m·K) [6,7].

Linear dimensions stability was tested by heating appropriately sized samples at 150 °C for 20 and 40 h. The tested compositions were characterized by good dimensional stability after 20 and 40 h of exposure to elevated temperatures (Table 3). Foams with the addition of EG 290 showed slightly higher shrinkage than those with melamine, EG 096, and without the addition of flame retardant (Table 3).

**Figure 2:**
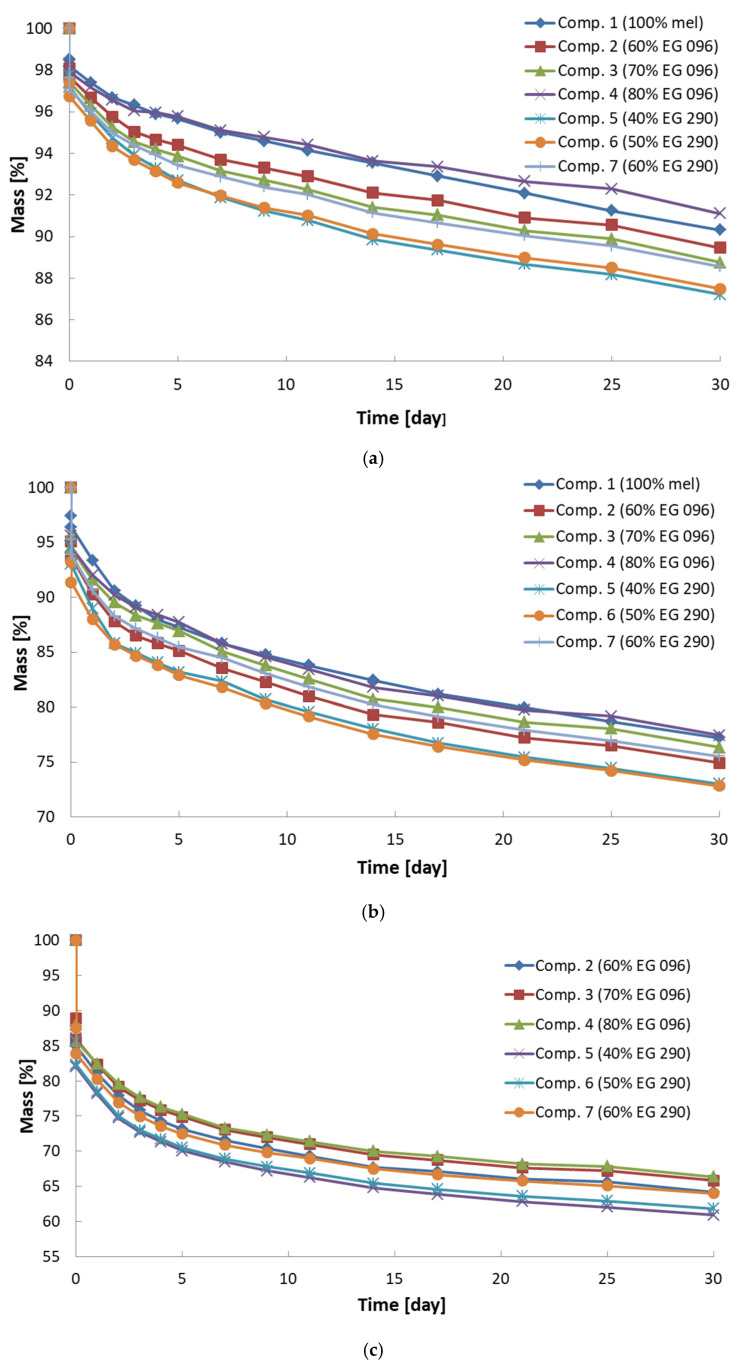
Thermal stability of polyurethane foams at temperature 150 °C (**a**), 175 °C (**b**), and 200 °C (**c**).

Static thermal resistance tests showed that the largest mass losses of the foams were observed during the first 2–3 days of heating (Figure 2a–c).

**Figure 3:**
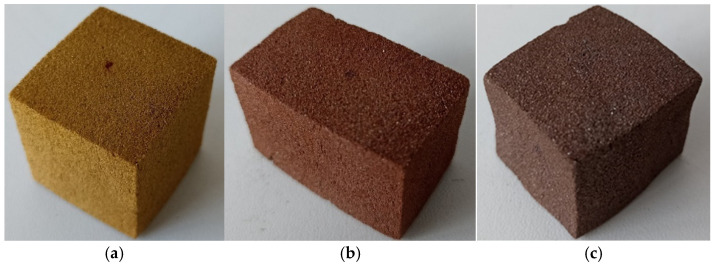
Composition 3 (70% EG 096) after exposure at a temperature of 150 °C (**a**), 175 °C (**b**), and 200 °C (**c**).

The composition with melamine as a flame retardant was significantly deformed after the first day of heating at 200 °C, so further exposure at this temperature was stopped. Samples heated at 150 °C and 175 °C underwent only slight deformation. The compositions with EG 096 did not deform after exposure at 150 °C and 175 °C but slightly deformed after exposure at 200 °C (Figure 3a–c).

**Figure 4:**
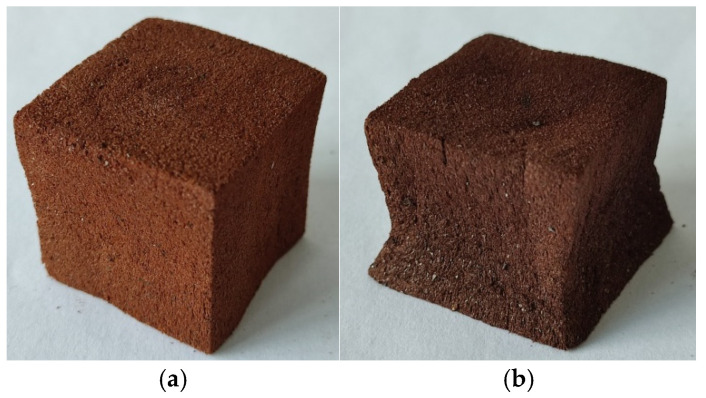
Composition 7 (60% EG 290) after exposure at a temperature of 175 °C (**a**) and 200 °C (**b**).

The compositions with the addition of EG 290 heated at 150 °C did not show any shape change; those heated at 175 °C underwent slight deformation; and those heated at 200 °C showed a greater deformation (Figure 4a,b).

Of the foams tested, composition 4, containing 80% EG 096, showed the lowest mass loss after a month of exposure at each temperature (150, 175, 200 °C). Comparing the mass losses of compositions containing the same amount of expanded graphite—compositions 2 (60% EG 096) and 7 (60% EG 290)—shows that they are very similar throughout the temperature range (Table 4). Therefore, the conclusion is that the thermal resistance of polyurethane foam is not affected by the type of expanded graphite, but only by the amount of this flame retardant. The addition of expanded graphite to a polyurethane foam composition increases its thermal resistance in direct proportion to the amount of this flame retardant. Foams containing expanded graphite in the amount of 60–80% in relation to the mass of the oligoetherol used had lower mass losses after 30 days of heating at 200 °C than foam without the addition of flame retardant (Table 4).

**Figure 5:**
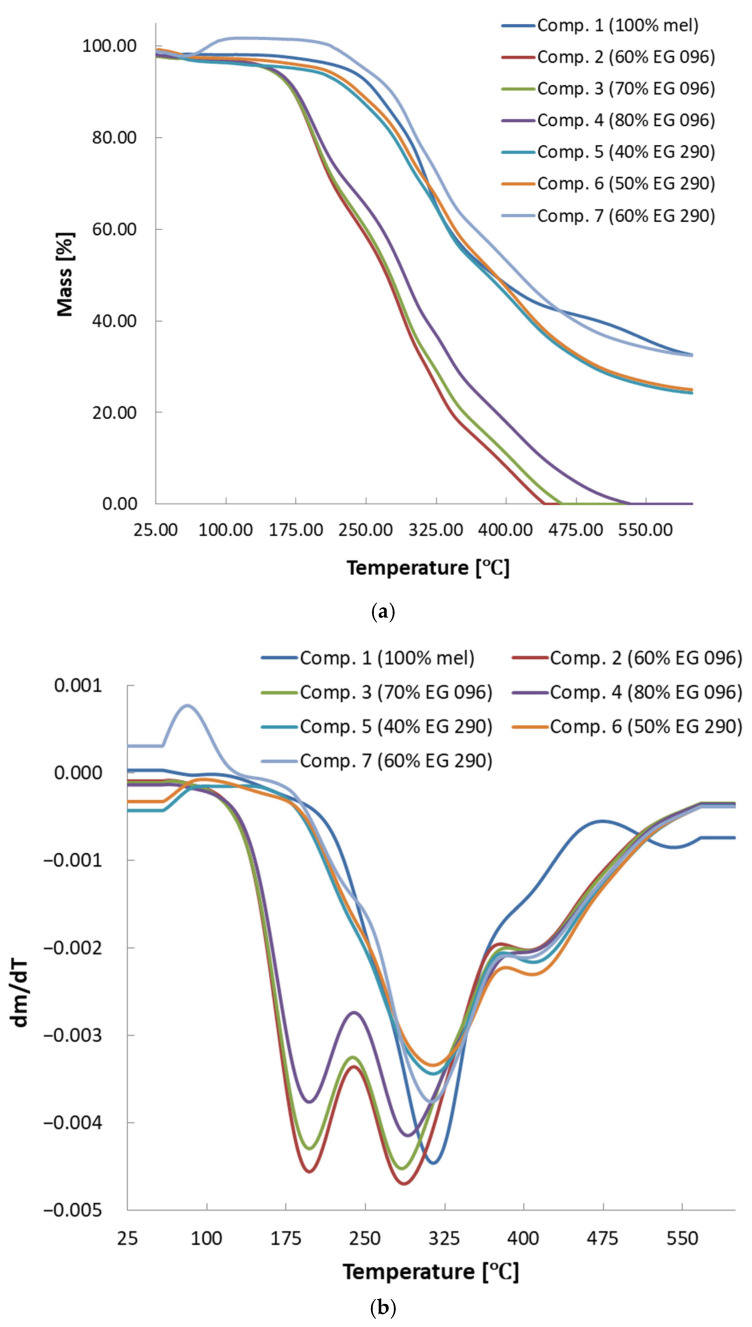
Thermal analysis of foams: TG (**a**) DTG (**b**) curves.

The good thermal resistance of the foams obtained is confirmed by dynamic thermal analysis (Table 4, Figure 5a,b). A 5% mass loss of the compositions is observed at temperatures from 161 to 249 °C and a 50% mass loss is observed in the range from 276 to 415 °C, while the maximum mass loss was recorded in the range of 285–315 °C (Table 4, Figure 5a,b). The differences in these values are related to the flame retardant used. Melamine can react with p-MDI, partially incorporating it into the foam structure. Furthermore, the 1,3,5-triazine ring is characterized by high thermal resistance, hence the 5% weight loss at 229 °C. Foams with a high concentration of graphite, probably due to the inhomogeneous dispersion of flame retardants, have a weakened structure, which causes degradation to begin at a lower temperature. A composition containing 60% EG 290 shows the highest degradation onset temperature (249 °C), suggesting the formation of a stable protective layer that effectively slows thermal degradation. The foams with melamine and EG 290 were not completely decomposed (Figure 5a, comp. 1 and 5–7), the pyrolysis residue of compositions 1 (100% melamine) and 7 (60% EG 290) was approximately 32%, and that of compositions 5 (40% EG 290) and 6 (50% EG 290) was about 25%.

On the DTG curves of foams with melamine and expanded graphite EG 290 (Figure 5b, comp. 1 and 5–7), essentially two endothermic peaks are observed at temperatures of around 315 °C and 410 °C. The first peak is related to the decomposition of polyurethane bonds into amine and carbon dioxide; the second is related to the dissociation of ether bonds [41,42]. On the DTG curves of foams with expanded graphite EG 096 (Figure 5b, comp. 2–4), an additional peak is observed at 190 °C, probably related to the thermal dissociation of the polyurethane bonds in the weakened foam structure. In the thermogravimetric test, foams with EG 290 and melamine as flame retardants showed better resistance than foams with the addition of EG 096 (Table 4). Foams with boron atoms as flame retardants were characterized by worse thermal resistance than the discussed materials [6,7].

The compressive strength of the composition was from 0.22 to 0.30 MPa (Table 5), which corresponds to the range for rigid polyurethane foams. It can be seen that the addition of melamine and expanded graphite in an amount of 40–70% compared with the mass of oligoetherol resulted in foams of higher strengths than the sample without flame retardant (Table 5). In the case of compositions with EG 096 and EG 290, their strength decreases with the increasing content of a given graphite; the lowest strength (0.22 MPa) was observed in the sample containing 80% EG 096. Exposure to temperature had a positive effect on the mechanical properties of the compositions. Heating the foams with the addition of expanded graphite for 30 days at 175 °C resulted in more than double their compressive strength (Table 5). The samples, after exposure to 200 °C, achieved the highest mechanical strength, with the highest increase shown by compositions containing EG 290 (Table 5).

**Figure 6:**
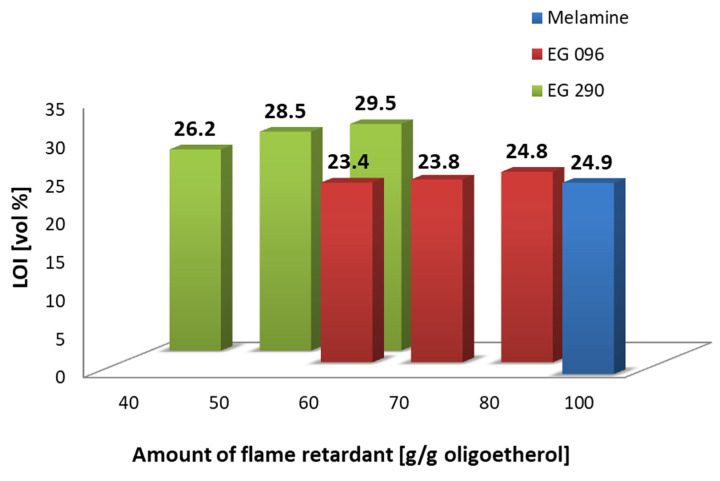
Oxygen index of polyurethane foams.

**Figure 7:**
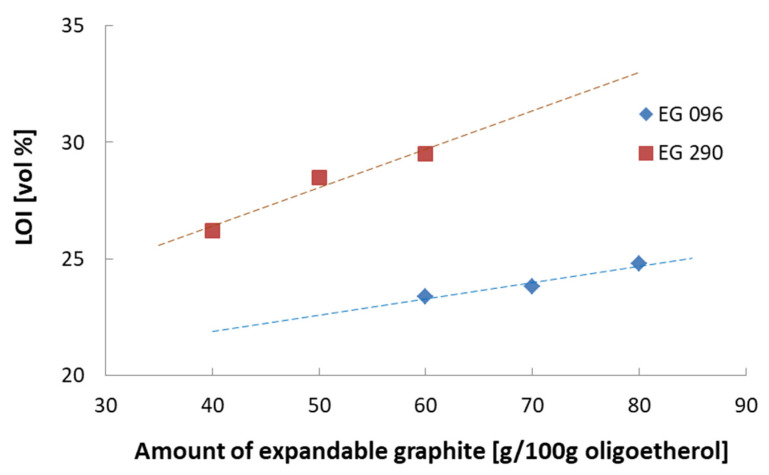
The dependence of the oxygen index on the amount of expanded graphite in the composition.

To determine the effect of melamine and expanded graphite on the flammability of the obtained materials, the oxygen index LOI [37] was determined and horizontal [38] and vertical [39] burning tests were performed. The oxygen index of the tested foams was in the range of 24.9–29.5 vol.% (Figure 6) and increased proportionally with the increasing content of both EG 096 and EG 290 (Figure 7). This is due to the thermal expansion of graphite, which creates a thermally insulating layer on the foam surface that prevents the spread of fire [19,25]. The highest oxygen index values (above 26.5) were shown by compositions with the addition of EG 290 (Figure 6). It can be seen that the introduction of EG 290 in the amount of 40% per 100 g of oligoetherol provides a higher flame-retardant effect than twice the amount (80%) of EG 096 (Figure 6). This proves that the expansion coefficient and particle size have a very large influence on the flame-retardant properties of expanded graphites [21].

**Figure 8:**
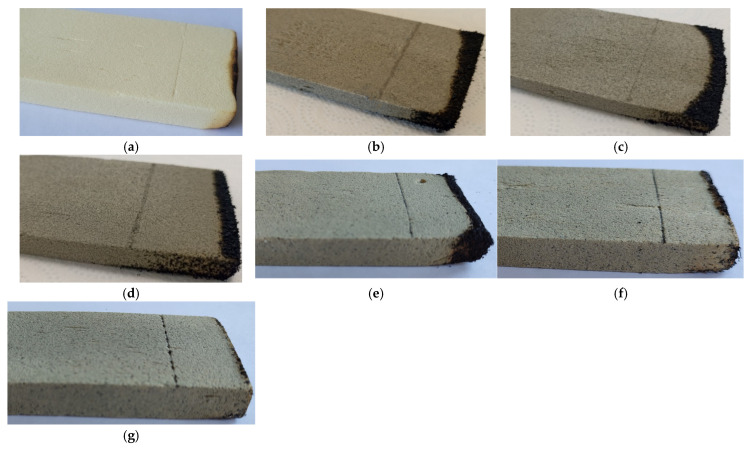
Horizontal burning test: composition no. 1 (**a**), composition no. 2 (**b**), composition no. 3 (**c**), composition no. 4 (**d**), composition no. 5 (**e**), composition no. 6 (**f**), and composition no. 7 (**g**).

Horizontal flammability tests showed that all foams burnt exclusively in the burner flame and were extinguished after the source of the flame (Table 6). In no case was the distance greater than 25 mm from the edge of the sample (Figure 8a–g). Composition 7, which contains the largest amount of EG 290, did not ignite at all in the burner flame; a minimal layer of char was formed on the foam surface (Figure 8g). The compositions tested can be classified as being of the HF-1 flammability class [38].

**Figure 9:**
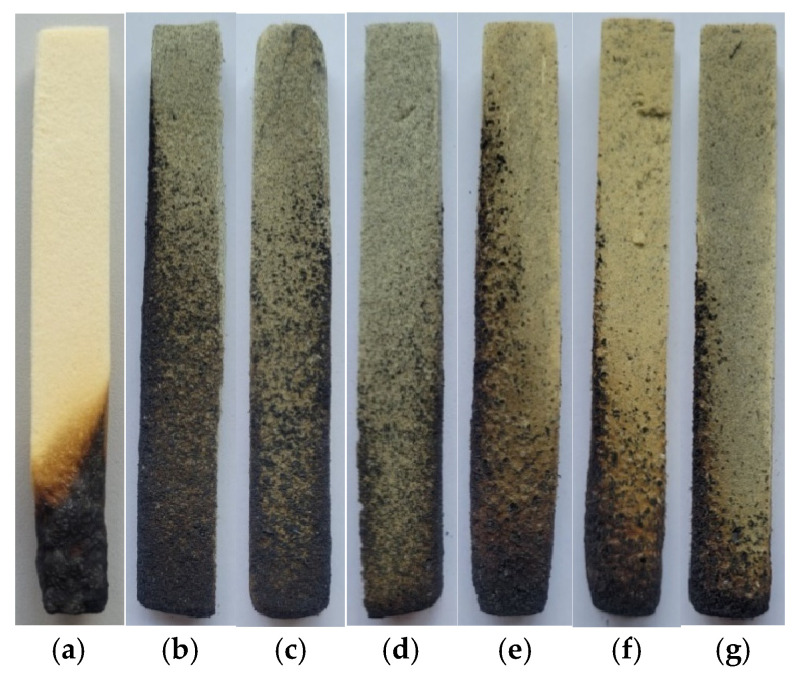
Vertical burning test: composition no. 1 (**a**), composition no. 2 (**b**), composition no. 3 (**c**), composition no. 4 (**d**), composition no. 5 (**e**), composition no. 6 (**f**), and composition no. 7 (**g**).

In the vertical flammability tests, the foams burnt exclusively in the burner flame and were extinguished after the flame was removed (Table 6). A char layer was formed on the surface of the foams with added graphite (Figure 9a–g). When reapplying the flame, these samples did not ignite because the char insulated the foam from the flame. All tested foams could be classified as being of the flammability class V-0, according to the vertical flammability test guidelines (Table 6) [39].

Based on the flammability tests carried out, foams with the addition of melamine and expanded graphite as a flame retardant can be classified as flame retardant and self-extinguishing. A composition containing 60% EG 290 is non-flammable and self-extinguishing [43].

## 4. Conclusions

The foaming of oligoetherol obtained by the reaction of 6-aminouracil, ethylene carbonate, and propylene oxide with polymeric diphenylmethane 4,4′-diisocyanate in the presence of water, triethylamine, and silicone and with the addition of melamine or EG 096 or EG 290 as a flame retardant leads to the formation of rigid polyurethane foams. The oxygen index of these materials is in the range of 24.9–29.5 vol.% and all of them belong to the flammability class V-0. A composition containing 60% EG 290 has an oxygen index of 29.5 vol.% and is non-flammable. Foams with the addition of melamine can be used at temperatures up to 175 °C, and with the addition of expanded graphite, up to 200 °C. The apparent density of the materials obtained is within the range of the apparent density of rigid foams. The addition of melamine or expanded graphite as a flame retardant does not negatively impact properties such as dimensional stability, water absorption, thermal conductivity, and compressive strength. These values are comparable to those of classic polyurethane materials. Expanded graphite (especially EG 290) is a better flame retardant than melamine and does not degrade the thermal resistance of the foams.

## Figures and Tables

**Table 1:** The influence of composition on the foaming process.

Flame Retardant	Composition Number	Composition [g/100 g Oligoetherol 6-AU:EC:PO = 1:5:3]					Foaming Process		
		Flame Retardant	p-MDI	Water	TEA	Silicon	Cream Time [s]	Growing Time [s]	Tack-Free Time [s]
Melamine	1	100	144	3	0.27	3.50	19	35	2
EG 096	2	60	144	3	0.61	3.84	24	32	1
	3	70	144	3	0.67	3.84	25	38	1
	4	80	144	3	0.94	3.84	23	30	1
EG 290	5	40	144	3	0.54	3.84	26	31	5
	6	50	144	3	0.54	3.84	26	31	5
	7	60	144	3	0.54	3.84	24	33	1

**Table 2:** Density, absorption of water, and thermal conductivity coefficient of polyurethane foams.

Composition Number (Amount and Kind of Flame Retardant)	Density [kg/m^3^]	Absorption of Water [vol.%]			Thermal Conductivity Coefficient λ [W/m∙K]
		After 5 min	After 3 h	After 24 h	
1 (100% melamine)	76	1.10	1.89	3.41	0.033
2 (60% EG 096)	66	0.70	2.10	4.10	0.034
3 (70% EG 096)	65	0.70	2.00	3.40	0.036
4 (80% EG 096)	60	0.90	1.50	2.40	0.037
5 (40% EG 290)	52	1.30	2.50	4.50	0.028
6 (50% EG 290)	51	1.60	2.50	3.90	0.033
7 (60% EG 290)	52	1.20	2.10	2.70	0.033
foam without flame retardant [29]	44	4.10	7.38	9.11	-

**Table 3:** Linear dimensions stability of polyurethane foams.

Composition Number (Amount and Kind of Flame Retardant)	Linear Dimensions Stability at Temperature 150 °C					
	Length Increase [%]		Width Increase [%]		Depth Increase [%]	
	After 20 h	After 40 h	After 20 h	After 40 h	After 20 h	After 40 h
1 (100% melamine)	−1.24	−1.55	−1.11	−1.24	−1.17	−1.45
2 (60% EG 096)	−0.37	−1.12	−1.11	−2.52	0.14	−0.20
3 (70% EG 096)	−0.49	−1.14	−0.66	−1.36	−0.43	−1.53
4 (80% EG 096)	−0.55	−1.10	1.29	−1.48	−0.54	−1.62
5 (40% EG 290)	−1.49	−2.12	−2.06	−2.47	0.30	−1.10
6 (50% EG 290)	−1.87	−2.24	−2.23	−2.97	0.81	−0.01
7 (60% EG 290)	−1.57	−2.34	−1.76	−2.77	0.84	0.14
foam without flame retardant [29]	0.00	−1.17	−0.05	−0.75	0.00	1.52

**Table 4:** Mass loss of polyurethane foams after 30 days of exposure to high temperature and thermal analysis.

Composition Number (Amount and Kind of Flame Retardant)	Mass Loss After 30 Days Exposure in Temperature [wt.%]			Thermal Analysis				
	150 °C	175 °C	200 °C	T_5%_ [°C]	T_10%_ [°C]	T_25%_ [°C]	T_50%_ [°C]	T_max_ [°C]
1 (100% melamine)	9.7	22.8	-	229	260	307	387	315
2 (60% EG 096)	10.5	25.1	35.8	161	177	207	276	290
3 (70% EG 096)	11.2	23.7	34.2	160	178	210	280	285
4 (80% EG 096)	8.9	22.5	33.6	162	180	218	295	290
5 (40% EG 290)	12.8	27.0	39.1	179	235	294	380	315
6 (50% EG 290)	12.5	27.2	38.2	202	242	300	388	315
7 (60% EG 290)	11.4	24.5	36.0	249	276	318	415	315
foam without flame retardant [29]	7.9	24.7	37.8	-	200	260	400	-

**Table 5:** Compression strength of polyurethane foams.

Composition Number (Amount and Kind of Flame Retardant)	Compression Strength			
	Before Exposition	After Exposition at		
	σ_10_ [MPa]	150 °C	175 °C	200 °C
		σ_10_ [MPa]	σ_10_ [MPa]	σ_10_ [MPa]
1 (100% melamine)	0.270	0.330	0.114	-
2 (60% EG 096)	0.280	0.320	0.610	0.780
3 (70% EG 096)	0.250	0.360	0.530	0.980
4 (80% EG 096)	0.220	0.260	0.510	0.810
5 (40% EG 290)	0.300	0.470	0.710	1.740
6 (50% EG 290)	0.250	0.430	0.700	1.430
7 (60% EG 290)	0.260	0.360	0.630	1.220
foam without flame retardant [29]	0.220	-	-	-

σ_10_—compression strength at 10% strain.

**Table 6:** Horizontal and vertical burning tests of polyurethane foams.

Composition Number (Amount and Kind of Flame Retardant)	Horizontal Burning Tests		Vertical Burning Test				
	Linear Burning Rate [mm/s]	Rating	Burning Time After First Flame Application [s]	Burning Time After Second Flame Application [s]	Combustion Up to Holding Clamp (Specimens Completely Burned)	Dripping of Burning Specimens, Ignition of Cotton Batting	Rating
1 (100% melamine)	the foam goes out immediately	HF-1	1	0	No	No	V-0
2 (60% EG 096)	the foam goes out immediately	HF-1	0	0	No	No	V-0
3 (70% EG 096)	the foam goes out immediately	HF-1	0	0	No	No	V-0
4 (80% EG 096)	the foam goes out immediately	HF-1	0	0	No	No	V-0
5 (40% EG 290)	the foam goes out immediately	HF-1	0	0	No	No	V-0
6 (50% EG 290)	the foam goes out immediately	HF-1	0	0	No	No	V-0
7 (60% EG 290)	the foam goes out immediately	HF-1	0	0	No	No	V-0

## Data Availability

The original contributions presented in the study are included in the article, further inquiries can be directed to the corresponding author.

## Abbreviations

The following abbreviations are used in this manuscript:

6-AU	6-aminouracil
EC	ethylene carbonate
EG	expanded graphite
LOI	limiting oxygen index
p-MDI	polymeric diphenylmethane 4,4′-diisocyanate
PO	propylene oxide
TEA	triethylamine
